# Grafting to Manage Infections of the Emerging Tomato Leaf Curl New Delhi Virus in Cucurbits

**DOI:** 10.3390/plants12010037

**Published:** 2022-12-21

**Authors:** Mariarosaria Mastrochirico, Roberta Spanò, Tiziana Mascia

**Affiliations:** Department of Soil, Plant and Food Sciences, University of Bari “Aldo Moro”, 70126 Bari, Italy

**Keywords:** begomovirus, mechanical inoculation, ToLCNDV, biodiversity, grafting-mediated virus resistance

## Abstract

Tomato leaf curl New Delhi virus (ToLCNDV) is an emerging begomovirus (*Geminiviridae* family) listed in the EPPO Alert List 2, present in the Mediterranean area and in Italy, where it was reported in 2015 in Sicilian courgette. The virus is widespread in cucurbits where it causes up to 100% production losses. In 2018, ToLCNDV was isolated in Apulia (southern Italy) in commercial fields of zucchini squash and since then its recurrent outbreaks generated justified concern among growers. Thus, a sustainable and environmentally friendly approach must be adopted. Genetic resistances have been identified in *Cucurbita moschata* and *Luffa cylindrica* but, compared to genetic resistance, grafting could provide a faster and more flexible solution because the graft wounding induces tolerance rather than resistance against airborne virus infection. Compared to tolerance, the up-regulation of resistance genes requires energy resources mobilized at the expense of primary metabolism, plant growth, and development. Results of screening among twenty-one local cucurbit cvs. ecotypes and accessions to evaluate tolerance levels against rub-inoculation of ToLCNDV led to the identification of potential rootstocks to attain suitable levels of tolerance against the virus in commercial cucurbit varieties. Cucurbit plants were challenged by a ToLCNDV isolated in Apulia denoted ToLCNDV-Le and evaluated for disease symptoms development and viral DNA accumulation up to 28 days after inoculation. On the basis of disease symptoms developed, plants were classified as tolerant, moderately tolerant, moderately susceptible, and susceptible. *Cucumis melo* cv. Barattiere did not show any detectable disease symptoms and very low levels of viral DNA accumulation was recorded; thus, it was used as rootstock for some of the remaining cucurbit genotypes that were used as scions. The tolerance trait was transmitted to the otherwise susceptible and moderately susceptible cucurbit genotypes grafted onto the cv. Barattiere. The results of this study suggest practical implications of the approach described.

## 1. Introduction

Tomato leaf curl New Delhi virus (ToLCNDV) is an emerging begomovirus (family *Geminiviridae*) for the Mediterranean area, including Italy, where it was first reported in 2015 in Sicilian courgette crops [1]. ToLCNDV was restricted to Asian countries until 2012 [2] when it was first reported in Spain for causing severe outbreaks in crops of the genus *Cucurbita* rather than in tomatoes [3]. For this reason, and for its eco-epidemiology, ToLCNDV is now listed as a quarantine pathogen on the Alert List 2 of the European and Mediterranean Plant Protection Organization (EPPO 2022; https://gd.eppo.int/taxon/TOLCND, accessed on 10 November 2022).

The virus is transmitted in a persistent manner by *Bemisia tabaci* [4], which probably exported it to Italy in colonies that infested strawberry seedlings from Spain [3], although recent evidence has also shown ToLCNDV transmission by seeds [5]. Beyond the name, the virus is particularly harmful to cucurbits grown in open fields and in greenhouses, where it can cause production losses close to 100%. Overall symptoms consist of severe curling and blistering of the apical leaves, which may also show loss of symmetry, mosaic, interveinal chlorosis, or necrosis; plants infected early are generally stunted (EPPO, 2017; [6]). This new situation caused growing concern in nurseries and farms since the cucurbit crops in use are routinely grafted onto rootstocks such as Schintoza F1, RS841 F1, Ercole, Vitalley, or OL1330 F1 that, considering the severity of disease observed in cucurbits grown in open fields and greenhouses, do not mitigate the effects of ToLCNDV infections.

The ToLCNDV genome consists of two DNA molecules called DNA A and DNA B [7] and, outside European conditions, it can also support the replication of alpha and beta satellites that can modify disease symptoms induced in host plants [8]. The analysis of ToLCNDV isolates reported from other geographic areas indicated that this virus has a highly heterogeneous population not attributable to a particular geographic area or group of host species, even though the isolates emerging in Spain seem to have evolved by recombination, adapting to infect cucurbits and fewer tomatoes [6,9]. The Italian isolates of ToLCNDV form two groups, one consisting of isolates found only in Italy while the other group clusters together Italian, Spanish, Tunisian, and Moroccan isolates [10]. The first record of ToLCNDV in commercial fields of zucchini squash (*Cucurbita pepo*) grown on the Ionian coast, on the border between Apulia and Basilicata (southern Italy) dates back to 2018 [11]. Since then, virus outbreaks are recurrently recorded in greenhouses of Brindisi and Lecce Provinces (Apulia, southern Italy).

The rapid spread of the virus in the Mediterranean area requires the urgent adoption of containment measures. The traditional control measures for ToLCNDV need to be based on sound epidemiological principles that include new phytosanitary approaches, crop rotation, host plant resistance, and chemical and biological control of the vectors [12]. Genetic resistances have been identified in *Cucurbita moschata* [13] and *Luffa cylindrica* but it appears that resistant commercial varieties are still limited to a few examples [14,15].

However, owing to their large population size and short generation time, viruses have the potential to quickly evolve and adapt to new genetic backgrounds under natural selection pressure [16] that is exacerbated by intensive production systems and climate changes. This leads to the continuous emergence of new species and resistance-breaking (RB) strains [17,18,19,20], which often compromise the utility of the introgression of resistance gene(s).

The European Green Deal and the ‘Farm to Fork’ strategies are very ambitious political initiatives with the general objective of achieving climate neutrality in Europe by progressive and complete decarbonization (‘Net-Zero’), by making food systems more sustainable, and at least a 50% reduction in the use of chemicals. Research programs aimed at mitigating the loss of biodiversity and at the identification of local varieties or ecotypes, which show characteristics of resistance or tolerance to the most harmful pathogens, are in line with the increasing attention on the safeguarding of local biodiversity since the characterization and use of autochthonous genetic resources appears crucial for the enhancement of local productions. Following this perspective, and compared to classical genetic resistance, grafting could provide fast and flexible responses to the emergence of RB viruses using the tolerance resulting from the combined contribution of the rootstock and graft. The combined effect has been demonstrated by tolerance to infection of three air-borne plant viruses considered among the 10 most harmful to horticultural crops in tomato varieties and commercial tomato hybrids when grafted onto themselves or onto the Manduria tomato ecotype [21,22,23]. Grafting could become of practical importance in vegetable crops to confer the scion tolerance traits to virus infection in a shorter time and with lower costs than traditional breeding. In a similar way, we undertook a study aimed at evaluating the resistance/tolerance levels of cultivated cucurbit crops grafted onto commercial cvs., local ecotypes, and accessions, to the ToLCNDV infection. If successful, this approach would permit the enhancement of local ecotype deployment, especially if they are at risk of extinction.

The screening of wild plants and local ecotypes is usually adopted to find additional sources of resistance or tolerance, as confirmed by current studies on sources of resistance to the tomato yellow leaf curl virus (TYLCV) in the tomato wild relative *Solanum habrochaites* [24]. In this study, cvs., ecotypes, and accessions of *Cucurbita maxima*, *C. moschata*, *C. pepo*, *Cucumis melo*, and *Lagenaria siceraria* were screened for susceptibility or tolerance to the rub-inoculation of an Apulian isolate of ToLCNDV, found in the Lecce Province and denoted ToLCNDV-Le. Some genotypes and cvs. proved highly susceptible whereas *C. melo* cv. Barattiere was highly tolerant to ToLCNDV-Le rub-inoculation. These characteristics led to its use as rootstocks for the more susceptible cvs. and genotypes tested.

## 2. Results

### 2.1. Evaluation of ToLCNDV-Le Symptoms in the Selected Cucurbit Genotypes

**Figure 1:**
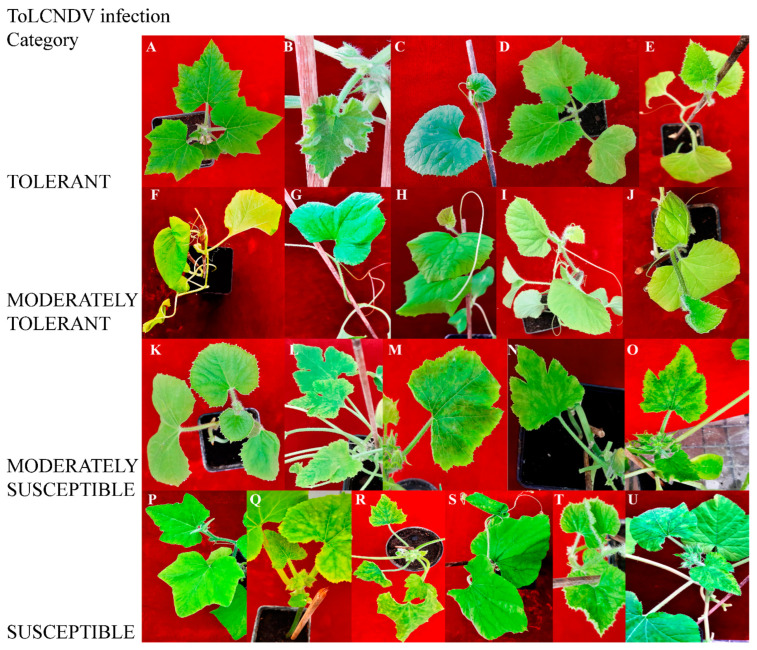
Systemic symptoms induced by ToLCNDV-Le at 28 dpi in different cucurbit species and cultivars classified as tolerant, moderately tolerant, moderately susceptible, and susceptible on the basis of disease symptoms developed: *C. maxima* cv. Invernale rigata (**A**); *C. pepo* accession 5 (**B**); *C. melo* cv. Tendral verde (**C**); *C. melo* cv. Barattiere (**D**); *C. melo* ecotype Invernale variopinto (**E**); *C. melo* ecotype Invernale giallo (**F**); *C. melo* ecotype Verde tondo (**G**); *C. melo* ecotype Invernale a fasce (**H**); *C. melo* ecotype Invernale bianco (**I**); *C. melo* cv. Rugoso di Cosenza (**J**); *C. melo* cv. Retato standard (no F1 hybrid) (**K**); *C. pepo* cv. President (**L**); *C. melo* ecotype Retato (Cantalupo) (**M**); *C. pepo* accession 63 (**N**); *C. moschata* cv. Moscata di Provenza (**O**); *C. moschata* cv. Cucuzza Genovese (**P**); *C. pepo* cv. Howden (**Q**); *C. pepo* cv. Scuro di Milano (**R**); *L. siceraria* spp. (**S**); *C. melo* cv. Retato standard (F1 hybrid) (**T**); *C. pepo* cv. Corritore (**U**).

In susceptible genotypes, symptoms of ToLCNDV-Le infection were reproduced and were similar to those observed in greenhouse infections, ranging from curling of the apical leaves to whole plant stunting. In symptomatic plants, new leaves lost symmetry, and showed mosaic, interveinal chlorosis, blistering, and curling of the leaf lamina, whereas other genotypes remained symptomless (Figure 1, Table 1 and Table S1).

In the first step of the assay, not all plants showed clear symptomatology since 6–8 out of the 10 rub-inoculated plants of *C. melo* ecotype Retato (Cantalupo), *C. pepo* cvs. Corritore and President, and accession 63 developed disease symptoms later than 10 dpi, and mild mosaic and moderate curling of the apical leaves were fully visible only by 28 dpi (Table 1 and Table S1). Therefore, these plants were classified as moderately susceptible on the basis of the symptoms category. Conversely, cultivars that did not show a delay in symptom onset and displayed leaf yellowing, curling, and stunting were considered susceptible cultivars (Table 1). These included *C. melo* cv. Retato standard (F1 hybrid); *C. pepo* cvs. Corritore, Howden, and Scuro di Milano; *L. siceraria* spp. *C. melo* ecotypes Invernale bianco and Invernale variopinto. *C. melo* cvs. Retato standard (no F1 commercial hybrid), Invernale giallo, Invernale a fasce, Rugoso di Cosenza, and Verde tondo showed very mild systemic symptoms and underwent recovery by 21 dpi so that no symptoms could be observed in the new vegetation at 28 dpi. Symptomless infections during the whole time period of the assay were observed in *C. maxima* cv. Invernale rigata, *C. melo* cv. Barattiere, *C. pepo* accession 5, and cv. Tendral (Table 1).

**Figure 2:**
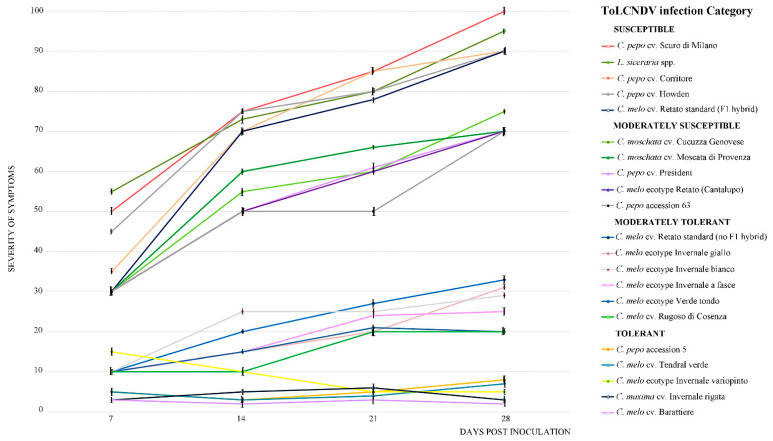
Severity of ToLCNDV-Le symptoms (from 0, absent, to 100, maximum severity, distributed in four equal parts) observed at 7, 14, 21, and 28 dpi in the 21-cucurbit genotypes infected by ToLCNDV-Le estimated according to the criteria reported in Table 1. Each point on the line chart represents average of ten plants of the same genotype showing the same symptom severity and error bars on lines represent the standard error among plants. Mock-inoculated plants served as negative control.

The severity of the ToLCNDV-Le disease phenotype (from 0, absence of symptoms, to 100, maximum symptoms severity, distributed in four equal parts) in all genotypes was also estimated at 7, 14, 21, and 28 dpi according to the criteria tolerant, moderately tolerant, moderately susceptible, and susceptible reported in Table 1 (Figure 2). By 7 dpi, all plants classified as susceptible displayed severe symptoms, which persisted throughout the experiment. These plants grouped together at the top of the graph and were clearly separated from plants classified tolerant or moderately tolerant (*C. melo* cv. Barattiere, Invernale variopinto, and Tendral verde; *C. maxima* Invernale rigata; *C. pepo* accession 5), which grouped together in the basal part of the graph. These plants showed no symptoms to very mild symptoms.

The presence of ToLCNDV-Le DNA was confirmed by dot blot analysis in systemically infected leaves at 28 dpi (Figure S1).

### 2.2. Viral DNA Accumulation in the Selected Cucurbit Genotypes

**Figure 3:**
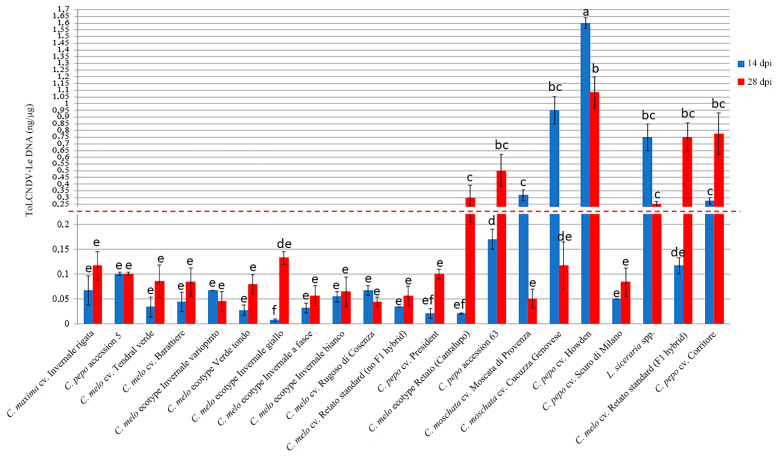
Relative dot-blot quantification of ToLCNDV-Le in systemically infected leaves of the selected 21 *Cucurbita* spp. at 14 (blue) and 28 (red) dpi. Virus DNA accumulation was assessed in two biological replicates of ten plants each, and two technical replicates by dot-blot hybridization by using a Digoxigenin-labeled DNA probe specific for ToLCNDV CP. The chemiluminescent signal was quantified by Quantity One software (BioRad Laboratories). Five dilutions (1:2) of a standard positive PCR product control were used. Statistically significant differences were calculated for each treatment and indicated with letters for *p* ≤ 0.05 using Tukey’s post hoc ANOVA test. Vertical bars on columns represent standard deviations among replicates.

Accumulation of viral DNA estimated in systemically infected leaf samples from two biological replicates of ten plants collected for each of the cucurbit species cvs. ecotypes and accessions at 14 and 28 dpi showed differences congruent with the disease symptom severity observed during the time-course analysis (Figure 3).

A significant increase in the accumulation of viral DNA between 14 and 28 dpi was observed in *C. melo* ecotype Retato (Cantalupo) (15-fold change, fc), *C. pepo* cv. Corritore (2.5 fc), *C. melo* cv. Retato standard (F1 hybrid) (6 fc) and *C. pepo* accession 63 (3 fc). Conversely, in the same time-point interval, a significant decrease in the accumulation of ToLCNDV-Le DNA was recorded in *C. moschata* cvs. Moscata di Provenza (fc -6), and Cucuzza Genovese (fc -8), in *L. siceraria* spp. (fc -3), and in *C. pepo* cv. Howden (fc -1.5). DNA of ToLCNDV-Le accumulated at very low levels in *C. melo* cvs. Rugoso di Cosenza, Retato standard (no F1 hybrid), and ecotypes Invernale bianco, Invernale giallo, Invernale a fasce, Invernale variopinto, and Verde tondo, which recovered from the mild disease symptoms between 21 and 28 dpi. *C. maxima* cv. Invernale rigata, *C. melo* accession 5, and cvs. Tendral verde and Barattiere did not recover from the very mild disease symptoms but showed very low levels of viral DNA accumulation as well, congruent with the mild symptomatology and the proposed identification as tolerant genotypes. Finally, the accumulation of viral DNA in *C. pepo* cvs. President and Scuro di Milano was very low at 14 dpi and increased only moderately between 14 and 28 dpi despite the two cvs. being classified as moderately susceptible (President) and susceptible (Scuro di Milano), respectively (Table 1 and Table S1).

### 2.3. Symptoms and Viral DNA Accumulation in Grafted Plants

Overall, *C. melo* cv. Barattiere and *C. pepo* accession 5 showed very low accumulation of ToLCNDV-Le DNA and were symptomless compared to the other species and cvs. Specifically, among the five different cucurbit genotypes that showed tolerance to viral infection, the *C. melo* cv. Barattiere was chosen as rootstock because it is a robust plant and has a stem diameter adaptable and compatible with that of the other plants used as scions.

**Figure 4:**
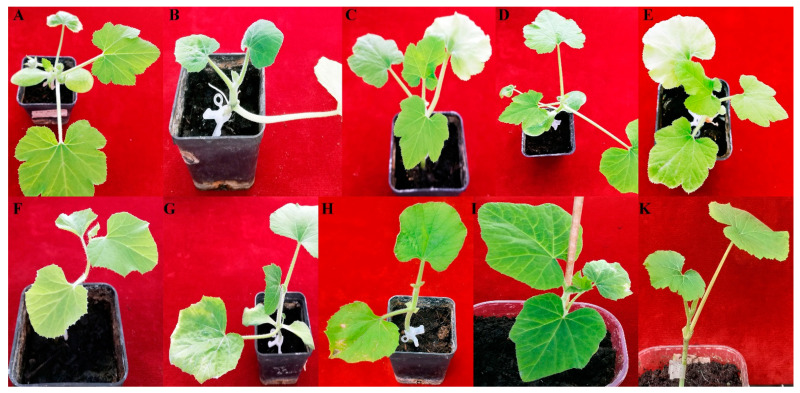
Disease phenotype induced by ToLCNDV-Le on the different graft combinations with susceptible and moderately susceptible cucurbit genotypes grafted on the tolerant local ecotype *C. melo* var. Barattiere used as common rootstock. Pictures taken at 14 dpi with ToLCNDV-Le: *C. pepo* cv. President (**A**); *C. moschata* cv. Moscata di Provenza (**B**); *C. pepo* cv. Howden (**C**); *C. pepo* cv. Scuro di Milano (**D**); *C. pepo* cv. Corritore (**E**); *C. melo* ecotype Retato (Cantalupo) (**F**); *C. moschata* cv. Cucuzza Genovese (**G**); *L. siceraria* spp. (**H**); *C. melo* cv. Retato standard (F1 hybrid) (**I**); *C. pepo* accession 6 (**K**).

On the basis of disease symptoms, viral DNA accumulation, and structural compatibility of the plant stem, scions of *C. moschata* cvs. Cucuzza Genovese and Moscata di Provenza, *C. melo* cv. Retato standard (F1 hybrid) and ecotype Retato (Cantalupo), *C. pepo* cvs. Corritore, President, Howden, Scuro di Milano, and accession 63, and *L. siceraria* spp. were grafted onto the cv. Barattiere (Figure 4 and Figure S2). In general, all grafted plants were robust and did not show suffering after grafting.

At an early stage of growth, rub-inoculation of ToLCNDV-Le did not influence the overall plant morphology and shape as these parameters were very similar to those of non-grafted plants but, at a later stage, grafted plants appeared less vigorous than non-grafted counterparts. On the whole, disease symptoms (Table 2) induced by virus infection on the scion of the different graft combinations showed poor variability but were less severe than those observed in non-grafted counterparts. In these plants, the ToLCNDV-Le disease phenotype consisted of mild systemic mosaic and leaf blade distortion and appeared between 14 and 28 dpi.

In addition, the number of grafted plants with detectable symptomatology was not very variable, ranging between 4 and 6 plants out of 10 grafted plants rub-inoculated (Table 3). A generalized delay in viral symptom appearance was observed in all graft combinations, suggesting that the grafting approach might improve cucurbits’ tolerance to ToLCNDV-Le infection.

**Figure 5:**
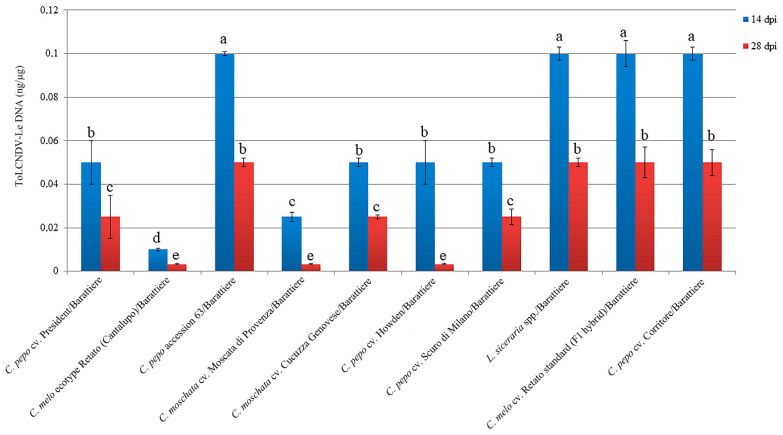
Viral DNA accumulation in systemically infected leaves of different genotypes infected with ToLCNDV-Le grafted onto *C. melo* cv. Barattiere used as rootstock. Viral DNA accumulation was estimated by quantitative dot-blot hybridization analysis by using a Digoxigenin-labeled DNA probe specific to the virus. Quantification of viral DNA at 14 (blue) and 28 (red) dpi was assessed by Image Lab software (BioRad Laboratories). Five dilutions (1:2) of a standard positive control were used. Different letters represent statistically significant differences of means according to factorial analysis of variance (ANOVA) (*p* ≤ 0.05) (Tukey’s test). Vertical bars on columns represent standard deviations among replicates.

The severity of disease symptoms in grafted plants was congruent with viral DNA accumulation estimated at 14 and 28 dpi. (Figure 5). A significant reduction in viral DNA accumulation was observed between 14 and 28 dpi in all grafted combinations. On the whole, the results from this screening suggest that cucurbit commercial varieties grafted onto *C. melo* cv. Barattiere used as rootstock could show interesting levels of tolerance against ToLCNDV-Le disease.

## 3. Discussion

In this study, we used rub-inoculation to evaluate the pathogenicity of the Apulian ToLCNDV-Le isolate in 21 cucurbit genotypes to identify a genotype with suitable levels of tolerance against this virus and potentially useful as a rootstock. In fact, *C. maxima* cv. Invernale rigata, *C. melo* cvs. Barattiere and Tendral Verde, and the ecotype Invernale variopinto were classified as tolerant on the basis of the mild disease symptomatology induced and viral DNA accumulation. Six additional genotypes of *C. melo* cvs. Rugoso di Cosenza and Retato standard (no F1 hybrid), and the ecotypes Invernale bianco, Invernale giallo, Invernale a fasce, and Verde tondo were classified as moderately tolerant according to the same criteria. The latter genotypes recovered from disease symptoms between 21 and 28 dpi so that they remained symptomless and with a very low level of viral DNA accumulation by the end of the assay. Recent evidences suggest that recovery from virus-induced disease symptoms might be the consequence of a robust delivery of secondary virus genome-derived siRNAs (vsiRNAs) generated by the host RNA interference (RNAi) mechanism to target and degrade viral genome in response to the infection. Begomoviruses are known to evade host-derived RNAi through suppressor proteins (VSRs) that, in the case of ToLCNDV, are encoded by AV2, AC2, and AC4 ORFs, which are ToLCNDV pathogenicity factors [6]. It has been proposed that VsiRNAs produced by the RNAi pathway saturate the viral suppressor of RNA silencing (VSRs) activity involved in determining disease symptoms with the establishment of a virus-tolerant condition in infected tissues [25,26].

ToLCNDV-Le infection induces very severe disease symptoms either in cucurbit crops grown in a field or some of the genotypes tested in this study. Fortes et al [9] analyzed the genetic variability of ToLCNDV isolates in Spain coming to the conclusion that the genetically homogeneous population of a new strain of ToLCNDV is present in Spain and is more efficient in infecting cucurbits species than tomatoes. Genetic analyses of these new strains suggested they putatively diverged by recombination from strains adapted to infect tomatoes [6,9]. Virulence of ToLCNDV-Le in cucurbits indicates it might be one of these new Spanish strains. Determination of the complete nucleic acid sequence of the ToLCNDV-Le genome is in progress in our lab and will clarify whether it arose from recombination or not and will determine its phylogenetic relationships with the other ToLCNDV so far sequenced.

The presence of ToLCNDV in the Mediterranean basin represents a new threat to economically important cucurbit crops and knowledge about the genetic diversity and host range of the isolates associated with the outbreaks is essential for durable control. Genetic resistance to ToLCNDV in *C. moschata* cvs. Large cheese and Indian landrace, classified as resistant [13], and in the accession BSUAL-252 originating from Japan, classified as highly resistant [13], have been identified and shown useful for introgression into commercial varieties [14,15]. In this study, two cvs. of *C. moschata* Moscata di Provenza and Genovese proved moderately susceptible. This discrepancy needs to be re-evaluated although it may be a trait of the cvs. used in this study. The use of ToLCNDV-resistant cultivars obtained by either conventional breeding or genetic transformation could indeed provide an effective way to minimize losses. However, the genetic uniformity of cucurbit plants in open fields might facilitate the emergence of virus isolates with better fitness to counteract the selection pressure exerted by the resistance gene on the viral population. In this scenario, the development of flexible and eco-sustainable antiviral strategies such as grafting seems affordable. In the case of soil-borne pathogens, it is well documented that grafting a susceptible scion onto a resistant rootstock provides a resistant plant without the need for a prolonged breeding program, allows for a more rapid response to the appearance of new races of the pathogen, and provides a less expensive and more flexible solution for controlling soil-borne diseases than breeding new resistant cultivars [27]. This approach has been used in Crete [28] and Spain [29] to protect muskmelons and watermelons against *Melon Necrotic Spotted Virus* (MNSV), which is a soil-borne virus but in tomatoes [23]; in this study, tolerance is conferred in grafted plants also against air-borne viruses. According to the recent literature [26,30], tolerant genotypes such as *C. melo* cv. Barattiere mitigate the impact of virus infection independently of virus load since they are refractory to virus accumulation and resilient to the appearance of disease symptoms. Indeed, plants of *C. melo* cv. Barattiere remained symptomless in all assays and with very low levels of viral DNA accumulation. Tolerance characteristics were substantially transferred to the otherwise susceptible cucurbit genotypes when grafted on the Barattiere cv. Of *C. melo*.

Results from this study are also in line with the Italian Recovery and Resilience Plan (RRP), and with European and national environmental targets in the field of sustainable agriculture and biodiversity.

## 4. Materials and Methods

### 4.1. Virus Source and Estimation of Virus Titre

The Apulian isolate ToLCNDV-Le was found in the Lecce Province (southern Italy) in greenhouse crops of zucchini squash (*C. pepo*) with strong symptoms of mosaic, yellowing, vein swelling, and severe curling of the leaf lamina. The virus was maintained and propagated in zucchini by rub-inoculation of leaves with the sap obtained from naturally infected tissues ground in Comav buffer (50 mM K-K_2_ potassium phosphate, pH 8.0, 1% polyvinylpyrrolidone 10, 1% polyethylene glycol 6000, 10 mM 2-mercaptoethanol, and 1% activated charcoal) in a proportion of 1:4 (*w*/*v*) [31].

Accumulation of ToLCNDV-Le DNA in infected plants was estimated at 14 and 28 dpi by quantitative dot-blot hybridization with a Digoxigenin-labeled (DIG) DNA-probe specific for the ToLCNDV gene coding for coat protein (CP), whereas the ToLCNDV-Le detection in moderately tolerant and tolerant cucurbit plants was performed in systemically infected leaves only at 28 dpi.

Total DNA was extracted from samples of leaves collected at the time points mentioned above, using the CTAB method [32]. Then, 3 μg of each DNA sample quantified with a Nanodrop 1000 spectrophotometer (Thermo Fisher Scientific, Italy) were mixed with 6 vol (*w*/*v*) of 50 mM NaOH and 2.5 mM disodium EDTA. The mixture was incubated at room temperature for 5 min and then 7 μL was spotted onto a positively charged nylon membrane (Roche Diagnostics, Mannheim, Germany) and exposed for 5 min to UV light to cross-link nucleic acids. Membranes were hybridized overnight at 50 °C in 150 μL/cm^2^ of DIG Easy HybGranules solution (Roche Diagnostics, Mannheim, Germany) containing 50 ng/mL of a DIG-labeled DNA probe derived from the KF891468 ToLCNDV sequence available in public database [10,33,34]. Both unlabeled DNA control and the corresponding DIG-labelled DNA probes were synthesized from 20 or 40 ng, respectively, of the same plasmid pCP-ToLCNDV-8 (kindly supplied by Prof. S. Davino of Department of Agricultural, Food and Forest Sciences SAAF, University of Palermo, Italy) preparation blotted onto a Whatman FTA card (Whatman FTA kit pK/1, GE healthcare life Sciences, Germany) and with PCR DIG Probe Synthesis Kit (Roche, Germany). PCR reactions were carried out using the following cycle: 3 min denaturation at 94 °C, followed by 35 cycles of 30 s denaturation at 94 °C, 30 s annealing at 55 °C, and 20 s synthesis at 72 °C, with the final elongation step for 5 min at 72 °C. Primer specificity was evaluated by checking PCR products in 1.2% agarose gel electrophoresis in TBE buffer (90 mM Tris, 90 mM boric acid, 1 mM EDTA) and gel-red staining. Primer pairs sequences used to amplify a Dig-DNA fragment of 1149 bp from recombinant plasmid containing the CP cloned segment of the ToLCNDV genome were forward-CP1 5′CTCCAAGAGATTGAGAAGTCC3′ and reverse-CP2 5′TCTGGACGGGCTTACGCCCT3′, as reported by Panno et al. [10] and Ruiz et al. [34]. Quantification of the viral DNA load was determined by using 1:2 serial dilutions in the alkaline solution of the quantified unlabeled DNA control and the gene *CpEF-1α* from *C. pepo* as an internal standard [13]. After hybridization, probe excess was removed by four washes of 15 min each with 2X SSC (300 mM NaCl, 30 mM Na citrate, pH 7) containing 0.1% SDS at 55 °C, followed by washes and hybrid detection according to the instructions of the DIG luminescent detection kit and CDP-star substrate (Roche Diagnostics, Mannheim, Germany). ChemiDoc™ Imaging System apparatus and Image Lab software (Bio-Rad Laboratories) were used to detect and quantify the chemiluminescent hybridization signal. Statistically significant differences for *p* ≤ 0.05 were assessed by one-way analysis of variance (ANOVA) with Tukey’s post hoc test, using Statistica software, version 7 (Stat Soft, Inc. 1984–2004).

### 4.2. Rub-Inoculation of ToLCNDV-Le onto Different Cucurbit Species and Grafting Procedure

Seedlings for each cv., ecotype or accession, were grown in a glasshouse at 22–25 °C with 18/6 light/dark photoperiod. Seedlings with 2–4 leaves were rub-inoculated with ToLCNDV-Le by grounding naturally infected leaves of zucchini squash in 1:4 (*w*/*v*) Comav buffer as described by López et al. [31]. Plants were monitored daily for symptom development.

The following local cucurbit ecotypes accessions and commercial cvs. were kindly provided by an Apulian nursery (Table 3) or selected from the DiSSPA-UNIBA germplasm collection (kindly provided by Prof. L. Ricciardi) to be tested for tolerance to ToLCNDV-Le: *Cucurbita. maxima* cv. Invernale rigata, *C. melo* cv. Barattiere, President, Retato standard, Retato standard F1 hybrid, Rugoso di Cosenza, and Tendral verde, as well as ecotypes Cantalupo, Invernale bianco, Invernale giallo, Invernale a fasce, Invernale variopinto, and Verde tondo; *C. moschata* cvs. Cucuzza Genovese and Moscata di Provenza; *C. pepo* cvs. Corritore, Howden, and Scuro di Milano and accessions 5 and 63; *Lagenaria. siceraria* spp.

On the basis of disease symptoms developed in response to ToLCNDV-Le infection, plants were classified as tolerant, moderately tolerant, moderately susceptible, and susceptible, according to the criteria proposed by Romero-Masegosa et al. [13] (Table 1).

The tolerant cv. Barattiere was used as rootstock to graft different ToLCNDV-Le susceptible and moderately susceptible genotypes: *C. moschata* cvs. Cucuzza Genovese and Moscata di Provenza, *C. melo* cv. Retato standard F1 hybrid, *C. pepo* cvs. Corritore, Howden, President, Scuro di Milano, accession 63 and *L. siceraria* spp. These genotypes were tested twice for susceptibility to ToLCNDV-Le prior to being used as scions in the grafting experiments onto rootstocks derived from the local ecotype *C. melo* cv. Barattiere. The grafting was carried out when seedlings had two to four true leaves and the stem diameter was 1.5–2 mm. A V-shaped and a T-shaped cut were adopted for the scion and the rootstock, respectively, which were clamped together with a silicon clip. After grafting, plants were covered with a polyethylene bag to reduce water losses by transpiration, as described by Spanò et al. [21]. After one week, the silicon clip was removed and grafted plants were rub-inoculated on the first leaf above the graft junction. Grafted and non-grafted plants were mock-inoculated with Comav buffer to serve as controls and monitored daily for symptom appearance. Ten biological replicates were prepared for each graft combination and treatment (mock or inoculated) and samples were collected from plants at 14 and 28 dpi for molecular analysis as described above.

## Figures and Tables

**Table 1:** Visual evaluation of symptoms induced by ToLCNDV-Le in *Cucurbita*, *Cucumis,* and *Lagenaria* spp. at 28 dpi classified as tolerant, moderately tolerant, moderately susceptible, and susceptible on the basis of disease symptoms developed.

ToLCNDV-Le Infection CATEGORY	SYMPTOMS	PHENOTYPE									
**TOLERANT**	No visible symptoms and normal growth	* 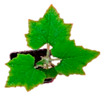 * *C. maxima* cv. Invernale rigata		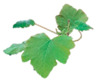 *C. pepo* accession 5		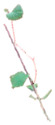 *C. melo* cv. Tendral verde		* 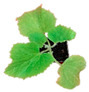 * *C. melo* cv. Barattiere		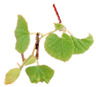 *C. melo* ecotype Invernale variopinto	
**MODERATELY** **TOLERANT**	Slight yellowing on leaves and normal growth	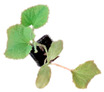 *C. melo* ecotype Verde tondo	* 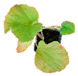 * *C. melo* Ecotype Invernale giallo		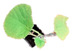 *C. melo* ecotype Invernale a fasce		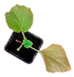 *C. melo* Ecotype Invernale bianco		* 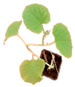 * *C. melo* cv. Rugoso di Cosenza		* 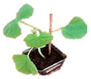 * *C. melo* cv. Retato standard
**MODERATELY** **SUSCEPTIBLE**	Yellowing and slight mosaic on leaves, normal plant growth	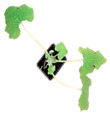 *C. pepo* cv. President		* 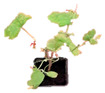 * *C. melo* ecotype Retato (Cantalupo)		* 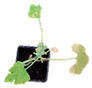 * *C. pepo* accession 63		* 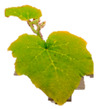 * *C. moschata* cv. Moscata di Provenza		* 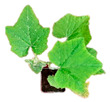 * *C. moschata* cv. Cucuzza Genovese	
**SUSCEPTIBLE**	Curling, yellowing and mosaic on leaves, stunted growth	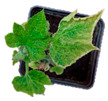 *C. pepo* cv. Howden		* 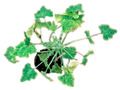 * *C. pepo* cv. Scuro di Milano		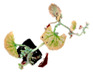 *L. siceraria* spp.		* 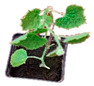 * *C. melo* cv. Retato standard (F1 hybrid)		* 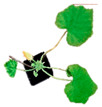 * *C. pepo* cv. Corritore	

**Table 2:** Averages of symptoms induced by ToLCNDV-Le infection on the scion of different plants grafted onto *C. melo* cv. Barattiere at 28 dpi.

Genotype	Symptoms ^1^	Grafted Plants with Symptoms ^2^
*Cucumis melo* cv. Retato standard (F1 hybrid)	MM; MLbD	5/10
*Cucurbita moschata* cv. Cucuzza Genovese	MM; MLbD	5/10
C. *moschata* cv. Moscata di Provenza	MM; MLbD	4/10
*Cucurbita pepo* accession 63	MM; MLbD	5/10
*C. pepo* cv. Howden	VMM; VMLbD	6/10
*C. pepo* cv. President	MM; MLbD	5/10
*C. pepo* cv. Scuro di Milano	VMM; VMLbD	4/10
*C. pepo* cv. Corritore	VMM; VMLbD	6/10
*C. melo* ecotype Retato (Cantalupo)	VMM; VMLbD	5/10
*Lagenaria siceraria* spp.	MM; MLbD	5/10

^1^ MM = mild mosaic; MLbD = mild leaf blade deformation; VMM = very mild mosaic; VMLbD = very mild leaf blade deformation. ^2^ No. of symptomatic plants out of 10 grafted plants rub-inoculated.

**Table 3:** List of different *Cucurbita genotypes* subjected to ToLCNDV-Le mechanical transmission.

*C. melo* ecotype Invernale bianco	*C. melo* ecotype Invernale a fasce	*C. melo* ecotype Invernale giallo	*C. melo* ecotype Retato (Cantalupo)	*C. melo* ecotype Verde tondo	*C. melo* cv. Tendral verde	*C. melo* ecotype Invernale variopinto
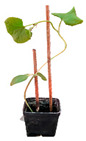	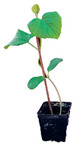	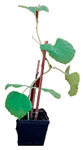	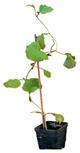	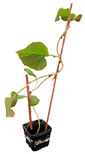	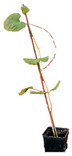	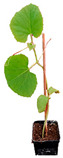
*C. melo* cv. Rugoso di Cosenza	*C. melo* cv. Retato standard (no F1 hybrid)	*C. melo* cv. Retato standard (F1 hybrid)	*L. siceraria* spp.	*C. pepo* cv. Scuro di Milano	*C. pepo* cv. President	*C. pepo* cv. Corritore
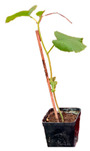	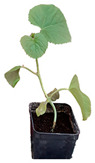	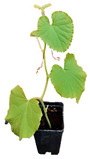	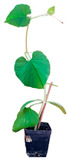	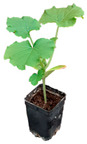	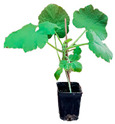	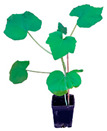
*C. pepo* accession 5	*C. pepo* accession 63	*C. pepo* cv. Howden	*C. moschata* cv. Moscata di Provenza	*C. moschata* cv. Cucuzza Genovese	*C. maxima* cv. Invernale rigata	*C. melo* cv. Barattiere
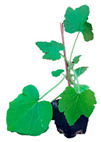	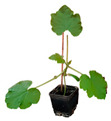	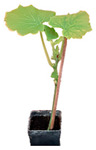	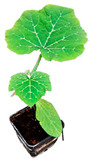	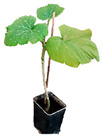	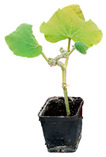	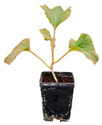

## Data Availability

All data, tables and figures in this manuscript are original.

## Supplementary Materials

The following supporting information can be downloaded at: https://www.mdpi.com/article/10.3390/plants12010037/s1, Figure S1: ToLCNDV-Le detection in moderately tolerant and tolerant cucurbit plants by Dot blot analysis from systemically infected leaves at 28 dpi: *C. melo* cv. Rugoso di Cosenza (**A**); *C. melo* cv. Retato standard (no F1 hybrid) (**B**); *C. melo* ecotype Invernale bianco (**C**); *C. melo* ecotype Invernale a fasce (**D**); *C. melo* ecotype Invernale giallo (**E**); *C. melo* ecotype Verde tondo (**F**); *C. maxima* cv. Invernale rigata (**G**); *C. pepo* accession 5; (**H**); *C. melo* cv. Tendral verde **(I**); *C. melo* cv. Barattiere (**J**); *C. melo* ecotype Invernale variopinto (**K**); C+ ToLCNDV PCR product served as positive control; C- Mock-inoculated plant served as negative control; Figure S2: List of the different graft-combinations of susceptible and moderately susceptible cucurbit genotypes grafted on the tolerant *C. melo* cv. Barattiere used as common rootstock. Pictures taken one week after grafting: *C. pepo* cv. President (**A**); *C. moschata* cv. Moscata di Provenza (**B**); *C. pepo* cv. Howden (**C**); *C. pepo* cv. Scuro di Milano (**D**); *C. pepo* cv. Corritore (**E**); *C. melo* ecotype Retato (Cantalupo) (**F**); *C. moschata* cv. Cucuzza Genovese (**G**); *L. siceraria* spp. *(***H**); *C. melo* cv. Retato standard (F1 hybrid) (**I**); *C. pepo* accession 6 (**L**); Table S1: Incidence of systemic disease symptoms induced by ToLCNDV-Le in 10 plants for each genotype at 14 and 28 dpi.
