# Supplementary material for: Grafting to Manage Infections of the Emerging Tomato Leaf Curl New Delhi Virus in Cucurbits
Source: Plants (Basel). 2022 Dec 21;12(1):37. doi: 10.3390/plants12010037 (PMC9824083; doi:10.3390/plants12010037)
Supplement: Supplementary file 1 [file plants-12-00037-s001.zip › Figure S1.pdf]

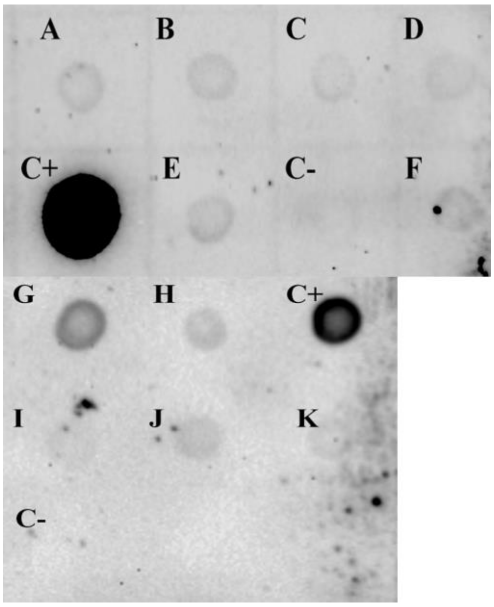

Figure S1. ToLCNDV-Le detection in moderately tolerant and tolerant cucurbit plants by Dot blot analysis from systemically infected leaves at 28 dpi: *C. melo* cv. Rugoso di Cosenza (**A**); *C. melo* cv. Retato standard (no F1 hybrid) (**B**); *C. melo* ecotype Invernale bianco (**C**); *C. melo* ecotype Invernale a fasce (**D**); *C. melo* ecotype Invernale giallo (**E**); *C. melo* ecotype Verde tondo (**F**); *C. maxima* cv. Invernale rigata (**G**); *C. pepo* accession 5; (**H**); *C. melo* cv. Tendral verde (**I**); *C. melo* cv. Barattiere (**J**); *C. melo* ecotype Invernale variopinto (**K**); C+ ToLCNDV PCR product served as positive control; C- Mock-inoculated plant served as negative control.
