# Supplementary material for: Grafting to Manage Infections of the Emerging Tomato Leaf Curl New Delhi Virus in Cucurbits
Source: Plants (Basel). 2022 Dec 21;12(1):37. doi: 10.3390/plants12010037 (PMC9824083; doi:10.3390/plants12010037)
Supplement: Supplementary file 1 [file plants-12-00037-s001.zip › Figure S2.pdf]

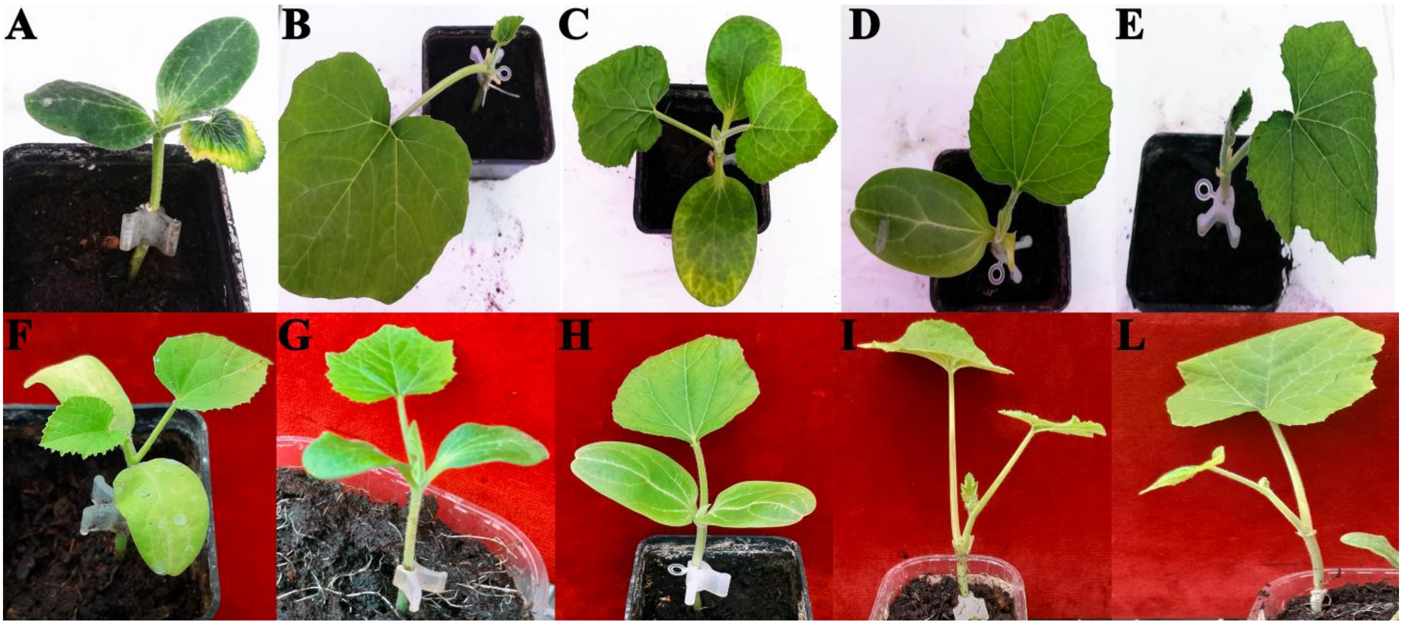

Figure S2. List of the different graft-combinations of susceptible and moderately susceptible cucurbit genotypes grafted on the tolerant *C. melo* cv. Barattiere used as common rootstock. Pictures taken one week after grafting: *C. pepo* cv. President (**A**); *C. moschata* cv. Moscata di Provenza (**B**); *C. pepo* cv. Howden (**C**); *C. pepo* cv. Scuro di Milano (**D**); *C. pepo* cv. Corritore (**E**); *C. melo* ecotype Retato (Cantalupo) (**F**); *C. moschata* cv. Cucuzza Genovese (**G**); *L. siceraria* spp. (**H**); *C. melo* cv. Retato standard (F1 hybrid) (**I**); *C. pepo* accession 6 (**L**).
