# Supplementary material for: Grafting to Manage Infections of the Emerging Tomato Leaf Curl New Delhi Virus in Cucurbits
Source: Plants (Basel). 2022 Dec 21;12(1):37. doi: 10.3390/plants12010037 (PMC9824083; doi:10.3390/plants12010037)
Supplement: Supplementary file 1 [file plants-12-00037-s001.zip › Table S1.pdf]

Table S1. Incidence of systemic disease symptoms induced by ToLCNDV-Le in 10 plants for each genotype at 14 and 28 dpi.

**Plants**	**Plants with Symptoms[1]**		**ToLCNDV-Le infection Category**
	**14 dpi**	**28 dpi**	
C. maxima cv. Invernale rigata	0/10	1/10	
C. pepo accession 5	0/10	0/10	
C. melo cv. Tendral verde	0/10	1/10	TOLERANT
C. melo cv. Barattiere	0/10	1/10	
C. melo ecotype Invernale variopinto	1/10	0/10	
C. melo ecotype Invernale giallo	1/10	1/10	
C. melo ecotype Verde tondo	1/10	1/10	
C. melo ecotype Invernale a fasce	1/10	1/10	MODERATELY TOLERANT
C. melo ecotype Invernale bianco	1/10	1/10	
C. melo cv. Rugoso di Cosenza	2/10	1/10	
C. melo cv. Retato standard (no F1 hybrid)	2/10	3/10	
C. pepo cv. President	7/10	10/10	
C. melo ecotype Retato (Cantalupo)	6/10	10/10	MODERATELY SUSCEPTIBLE
C. pepo accession 63	8/10	10/10	
C. moschata cv. Moscata di Provenza	10/10	10/10	
C. moschata cv. Cucuzza Genovese	10/10	10/10	
C. pepo cv. Howden	10/10	10/10	
C. pepo cv. Scuro di Milano	10/10	10/10	
L. siceraria spp.	10/10	10/10	SUSCEPTIBLE
C. melo cv. Retato standard (F1 hybrid)	8/10	10/10	
C. pepo cv. Corritore	8/10	10/10	

[1] No. of symptomatic plants out of 10 plants rub-inoculated.
